# Moyamoya Syndrome in Schimke Immune-Osseous Dysplasia: A Rare Association

**DOI:** 10.7759/cureus.25838

**Published:** 2022-06-10

**Authors:** Manoj Nayak, Biswamohan Mishra, Shailesh B Gaikwad, Kalyan Sarma, Manjari Tripathi

**Affiliations:** 1 Neuroradiology, All India Institute of Medical Sciences, New Delhi, IND; 2 Neurology, All India Institute of Medical Sciences, New Delhi, IND

**Keywords:** stroke, smarcal1, spondylo-epiphyseal dysplasia (sed), moya-moya disease, schimke immuno-osseous dysplasia (siod)

## Abstract

Schimke immuno-osseous dysplasia (SIOD) is an uncommon autosomal recessive (AR) spondylo-epiphyseal dysplasia (SED) and its clinical course and phenotype are yet to be properly described. The phenotypic presentation is quite varied with involvement of the renal, skeletal, vascular, immune, and hematopoietic systems being the most common presentation. We describe a 19-year-old female who presented with adolescent-onset brain and skeletal involvement without renal manifestations. Based on imaging and clinical features, she was diagnosed with a case of SIOD. There is no definitive treatment yet for this disorder, however, clinicians should be aware of this disorder so that adequate counseling and symptomatic management, especially in controlling hypertension and dyslipidemia, can be provided to the affected patients.

## Introduction

Schimke immuno-osseous dysplasia (SIOD) is an uncommon autosomal recessive (AR) spondylo-epiphyseal dysplasia with an estimated prevalence of 1 in 1-3 million [1]. Variable clinical manifestations are seen in SIOD with frequent involvement of the renal, skeletal, vascular, immune, and hematopoietic systems [2]. These patients will have a higher incidence of bone marrow hypoplasia, cerebral ischemia, and thyroid dysfunction with progressive renal failure [2]. Stroke is the major cause of morbidity and mortality in these patients [2]. Herein, we describe a 19-year-old female who presented to our department with brain and skeletal involvement without renal manifestations.

## Case presentation

A 19-year-old female presented with acute onset headache, recurrent vomiting, and behavioral disturbances for last 20 days, stiffness in right half of body, and facial deviation to left side 10 days back. She was a first-born child of non-consanguineous marriage having normal birth and developmental history. At five years of age, she complained of difficulty in walking after one episode of fever following which a shorter left lower limb was observed. A year later she complained of insidious onset progressive vision loss in left eye which progressed to complete vision loss over next three months followed by a similar complaint in the right eye within one month after involvement of first eye, for which she joined the blind school. She received physiotherapy for walking difficulty and was able to walk with support after two weeks. She was completely blind and her walking difficulty remained static during this period. Her cognition was normal. Examination revealed short stature with broad thorax, bilateral corneal opacities, and clinodactyly. Cranial nerve examinations were normal except for no perception of light in both eyes. The bulk of the muscles was normal with increased tone in both upper and lower limbs, and power could not be assessed. Her vitals were normal. Her routine hemogram, biochemical, metabolic, and autoimmune panel were within normal limits (Table 1).

**Table 1:** Results of the investigations performed on this patient at the time of admission Laboratory investigation results include hematological, biochemical, metabolic, and cerebrospinal fluid studies performed on this patient at the time of admission. CSF- cerebrospinal fluid, DLC- differential leukocyte count, RBC- red blood cell, LDL- low-density lipoprotein, HDL-high density lipoprotein, VLDL- very-low-density lipoprotein, CPK- creatinine phosphokinase, ANCA- anti-neutrophil cytoplasmic antibody, ANA- antinuclear antibody, TSH-thyroid stimulating hormone

Tests, units	At admission	Normal Range
Haemoglobin, g/100ml	9.2	12-18
Total leukocyte count, 10^3^/ml	6.58	5.20-12.40
Differential count,%		
Neutrophil	66.3	40-74
Lymphocyte	21.7	19-48
Monocyte	10.3	3.4-9.0
Eosinophil	0.8	0.00-7.0
Platelet count, 10^3^/ml	427	130-400
Urea, mg%	12	15-50
Creatinine, mg%	0.4	0.5-1.2
Sodium, meq/L	135	136-146
Potassium, meq/L	4.03	3.5-5
CSF		
Glucose, mg/dl	61	50-80
Protein, mg/dl	19	15-45
DLC	Nil	
RBC	340	
Culture	Sterile	
Zn staining	Negative	
Anti-ds DNA, IU/ml	5	0-100
Lipid profile		
Triglyceride, mg/dl	44	50-150
LDL, mg/dl	102	0-130
HDL, mg/dl	36	40-60
VLDL, mg/dl	14	10-30
TC, mg/dl	152	100-200
CPK,U/L	62	40-226
RF,IU/ml	Negative	
ANCA, IU/ml	Negative	
Anti Hep 2, IU/ml	Negative	
TSH,IU/ml	1.79	0.4-4
Vitamin D3, ng/ml	8.87	30-100
Vitamin B12, pg/ml	477	174-878

**Figure 1:**
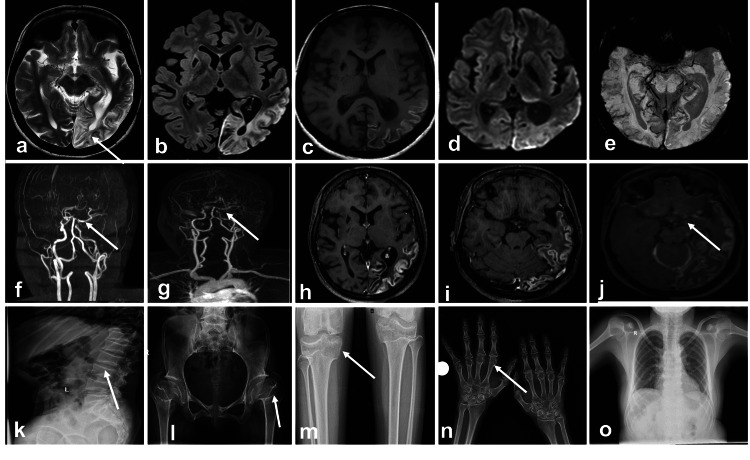
MRI brain and X-ray of the skeletal system of SIOD Axial T2 (a) and FLAIR (b) MRI images showed a hyperintense lesion in the left temporo-parieto-occipital lobe with gyriform T1 hyperintensity on T1 images (c). No restricted diffusion was diffusion seen on DWI images(d) and without blooming on SWI images(e). T2 hyperintense lesions with FLAIR suppression (b) were also seen in the right corpus striatum and posterior part of the right putamen s/o chronic infarcts. On TOF COW (f) and Magnetic resonance angiography (g), non-visualization of both supraclinoid internal carotid arteries (left>right) with mild proximal narrowing of the left internal carotid artery and prominent basal and pial collaterals. On post-contrast axial (h), enhancement of the infarct was seen. On vessel wall imaging pre (i) and post (j), narrowing with negative remodeling was seen in both supraclinoid ICA with a minimal enhancement of vessel wall imaging. Lateral view of lumbar spine X-ray (k) showed decreased posterior vertebral body height in lumbar and visualized thoracic vertebral bodies with thin ribs. AP view of pelvis X-ray (l) showed flattening of both femoral epiphyses with broadened iliac crest and pubic diastasis. AP view of both knee X-rays (m) showed flattening of both tibial epiphyses. X-ray of both hands (n) showed epiphyseal beaking in both metacarpals. X-ray chest PA view (o) showed thin ribs with a broad chest.

Magnetic Resonance Imaging (MRI) brain showed chronic infarcts with cortical laminar necrosis in the left temporoparietal lobe, chronic infarcts in the right corpus striatum, and posterior part of the right putamen, and chronic watershed infarct in the right posterior frontal lobe. On Magnetic Resonance Angiography (MRA), there was non-visualization of both supraclinoid internal carotid arteries (left>right) with mild narrowing of the left internal carotid artery. Vessel wall imaging revealed narrowing in both supraclinoid ICAs with contrast enhancement of the vessel walls. Multiple basal and pial collaterals were also noted (Figures 1a-1j). The short stature of the patient prompted a skeletal survey. Lumbar spine X-ray showed decreased posterior vertebral body height in lumbar and visualized thoracic vertebral bodies. Pelvis X-ray showed flattening of both femoral epiphyses with broadened iliac crest and pubic diastasis and knee x-ray showed flattening of both tibial epiphyses. X-ray chest showed thin ribs with a broad chest and the hand X-ray showed epiphyseal beaking in both metacarpals (Figures 1k-1o). These findings were consistent with spondylo-epiphyseal dysplasia with moyamoya syndrome (Schimke immune-osseous dysplasia). Parents were counseled regarding the need for genetic testing but declined for the same due to cultural issues.

The patient was started on tablet aspirin 75mg once daily, and tablet quetiapine 50mg twice daily. There was no further worsening during hospitalization. She was discharged and advised to follow up. At six months follow-up patient was doing well without headache, and improvement in her behavioral symptoms. Biochemical and metabolic parameters were within normal limits. She was continued on tablet aspirin 75mg once daily and was kept on close follow-up, and a plan of ECA-MCA (external carotid artery-middle cerebral artery) bypass has been kept in place if she suffers further stroke on aspirin.

## Discussion

Schimke immuno-osseous dysplasia (SIOD) is an uncommon AR disorder due to mutations in the *SMARCAL1* gene, which encodes for chromatin-remodeling enzyme necessary for stabilizing stalled replication forks, restricting DNA damage due to its replication [1]. The vascular changes in this disorder are due to disorganized elastogenesis with reduced elastin expression within the vessel, resulting in the defective internal elastic lamina and hyperplasia of intima or media [3]. This disorder manifests as spondylo-epiphyseal dysplasia (SED), immunodeficiency, growth retardation, and nephropathy. Neurologic features are microcephaly, seizures, stroke, intellectual disability, migraine-like headaches, and transient neurological attacks [1]. Corneal opacity was seen in 7/26 patients in one series [2].

Cerebrovascular disease management is vital to better life expectancy in patients due to the widespread availability of dialysis and renal transplantation [4]. Currently, no definite treatment is available for SIOD [4]. Adequate control of hypertension and hyperlipidemia with prevention of stroke is essential for which MRI brain with angiography can be done at regular intervals [4]. Antiplatelet/antithrombotic prophylaxis can be considered, with early and prompt management of dehydration and infection. Some patients with high-grade stenosis of intracranial vessels may be candidates for ECA-MCA bypass surgery [4].

The present case showed a varied phenotypic manifestation, with a relatively later age of presentation. Patients with severe disease present in the neonatal period with growth failure and death occur within the first 2-5 years, while milder disease present between 8-13 years of age with renal dysfunction and growth failure, which may progress to renal failure in the next 6-12 years [2]. Milder disease survives beyond 15-16 years and is usually spared from recurrent infections, bone marrow failure, and hypothyroidism [5]. The primary causes of mortality include infection (23%), stroke (17%), congestive heart failure and pulmonary hypertension (15%), renal failure (15%), organ transplantation complications (9%), lymphoproliferation (9%) gastrointestinal bleeding (6%), restrictive lung disease (3%), and bone marrow failure (3%) [6]. Boerkoel et al. have reported the largest cohort to date, wherein all the 39 patients had renal manifestations and growth disturbance with variable affection of other systems [2]. In the present case, there was though the involvement of the brain and skeletal system associated with growth failure at presentation, the renal system was spared to date, which is rare for this disease. Imaging usually shows narrowing of the aorta, internal carotid, and intracranial vessels with moyamoya-like disease [2] and very rare association like reversible cerebral vasoconstriction syndrome (RCVS) [7]. Likewise, the present case also had a moyamoya-like disease.

## Conclusions

SIOD is a rare disorder with varied phenotypic manifestations. In a patient with spondylo-epiphyseal dysplasia and stroke, Schimke immuno-osseous dysplasia to be kept in mind. A high degree of suspicion may help in early diagnosis and appropriate management may prevent from life-threatening complications, especially stroke. Regular follow-up is required to timely intervene in complications and improve the patient’s quality of life.
